# Abstract of Proceedings of the Buffalo Medical Association

**Published:** 1866-11

**Authors:** T. M. Johnson

**Affiliations:** Sec’y.


ART. III—*Abstract of Proceedings of the Buffalo Medical Association.*

TUESDAY EVENING, October 2d, 1866.

The meeting was called to order at the usual hour by the President. Members present—Drs. Gould, Samo, Wetmore, Cronyn, J. R. Lothrop, Abbot, Miner and Johnson.

The minutes of the last meeting were read and adopted.

Dr. J. R. LOTHROP related a case of alleged mistake by an apothecary in Lockport in putting up a prescription. Dr. Clark, of Lockport, the prescribing physician, states that he prescribed fld. ext. uva ursi in doses of about 12 drops, but that the apothecary put up fld. ext. veratrum viride, of which the patient took two doses, and was soon after taken with severe retching, difficult breathing, and soon became covered with a profuse cold sweat, and nearly pulseless, and exhibiting a death-like palor of the face. These symptoms were within a few hours followed by a severe attack of dysentery, which continued for several days. The patient sued the apothecary and obtained a verdict of $800 damages.

Dr. Lothrop has written a full history of the case, which is published in the *Buffalo Medical and Surgical Journal* of October.

Dr. JOHNSON related a case of alleged mistake in putting up a prescription by an apothecary in Chautauqua county. Was called in June last into said county to make a post mortem examination upon the body of woman whose death was claimed by the attending physician to have been caused by two doses of veratrum viride. The history of the case as ascertained from those most interested, was as follows:—The patient, a woman about fifty years of age, whose health had been quite infirm for two or three years, had, during the last three or four months of her life been more unwell than usual. Sometime in the month of April last her attending physician had prescribed for her Tilden's fld. ext. valerian to be taken in doses of 15 drops every four hours. The medicine was put up and properly labeled by the apothecary. About the last week in the following month the attending physician again prescribed the valerian, to be given in the same manner as formerly. The same bottle with the label upon it was then sent to the same apothecary, and by mistake filled with Tilden's fld. ext. veratrum viride. The patient not heeding the directions took about 30 drops of the medicine, and was about two hours after taken with considerable pain in the stomach, nausea and vomiting. From four to six hours after taking the first dose she took another, and still larger one—probably about 45 drops. This was within two hours followed by severe pain in the epigastrium, retching, vomiting, weak and rapid pulse, and marked prostration. Within 12 hours after taking the last dose she had two or three bloody stools with considerable tenesmus. Diarrhœa continued a few hours after the subsidence of the dysenteric discharges. The retching and vomiting continued at intervals for about four weeks, when she died. During this time but very little food was long enough retained to nourish the system in the least.

On post mortem examination all the viscera were found in normal condition except that the stomach was much less in size than is usually found in a medium sized female. This condition of the stomach was not, by either of the seven medical men present, attributed to the effects of the veratrum. The case will probably come before the courts, when all the facts it is to be hoped will be brought out.

Considerable informal interchange of sentiment was had upon the properties and effects upon the system of veratrum viride. There seemed to be pretty general agreement upon these points, that the most marked and peculiar influence of veratrum viride is its action upon the pulse. That it is, in other words, an actual sedative, and that vomiting although not invariably an effect following its administration, yet almost always occurs when it has been given in considerable doses; and that its peculiar sedative influence upon the pulse is more marked after vomiting occurs. That purging sometimes occurs after it has been given, but that it is in no proper sense a purgative. An ordinary emetic, as for instance ipecacuanha, is often followed by considerable purging. Therefore if purging follows the giving of veratrum viride, there is every reason to suppose that its operation in this particular is in no way different from that of ordinary emetics.

There were no especial diseases reported as prevailing. Adjourned. T. M. JOHNSON, Sec'y.

